# Mild episodes of tourniquet-induced forearm ischaemia-reperfusion injury results in leukocyte activation and changes in inflammatory and coagulation markers

**DOI:** 10.1186/1476-9255-4-12

**Published:** 2007-05-30

**Authors:** Stephen F Hughes, Beverly D Hendricks, David R Edwards, Salah S Bastawrous, Gareth E Roberts, Jim F Middleton

**Affiliations:** 1 Chemical Pathology Department, Glan Clwyd Hospital, Sarn Lane, Rhyl, Denbighshire, UK; 2 Haematology Department, Glan Clwyd Hospital, Sarn Lane, Rhyl, Denbighshire, UK; 3 Haematology Department, Gwynedd Hospital, Penrhosgarnedd, Bangor, Gwynedd, UK; 4 Orthopaedics Department, Glan Clwyd Hospital, Sarn Lane, Rhyl, Denbighshire, UK; 5 Anaesthetics Department, Gwynedd Hospital, Penrhosgarnedd, Bangor, Gwynedd, UK; 6 Leopold Muller Arthritis Research Centre, School of Medicine, Keele University at Robert Jones and Agnes Hunt Orthopaedic Hospital, Oswestry, UK

## Abstract

**Background:**

Monocytes and neutrophils are examples of phagocytic leukocytes, with neutrophils being considered as the 'chief' phagocytic leukocyte. Both monocytes and neutrophils have been implicated to play a key role in the development of ischaemia-reperfusion injury, where they are intrinsically involved in leukocyte-endothelial cell interactions. In this pilot study we hypothesised that mild episodes of tourniquet induced forearm ischaemia-reperfusion injury results in leukocyte activation and changes in inflammatory and coagulation markers.

**Methods:**

Ten healthy human volunteers were recruited after informed consent. None had any history of cardiovascular disease with each subject volunteer participating in the study for a 24 hour period. Six venous blood samples were collected from each subject volunteer at baseline, 10 minutes ischaemia, 5, 15, 30, 60 minutes and 24 hours reperfusion, by means of a cannula from the ante-cubital fossa. Monocyte and neutrophil leukocyte sub-populations were isolated by density gradient centrifugation techniques. Leukocyte trapping was investigated by measuring the concentration of leukocytes in venous blood leaving the arm. The cell surface expression of CD62L (L-selectin), CD11b and the intracellular production of hydrogen peroxide (H_2_O_2_) were measured via flow cytometry. C-reactive protein (CRP) was measured using a clinical chemistry analyser. Plasma concentrations of D-dimer and von Willebrand factor (vWF) were measured using enzyme-linked fluorescent assays (ELFA).

**Results:**

During ischaemia-reperfusion injury, there was a decrease in CD62L and an increase in CD11b cell surface expression for both monocytes and neutrophils, with changes in the measured parameters reaching statistical significance (p =< 0.05). A significant decrease in peripheral blood leukocyte concentration was observed during this process, which was measured to assess the degree of leukocyte trapping in the micro-circulation (p =< 0.001). There was an increase in the intracellular production of H_2_O_2 _production by leukocyte sub-populations, which was measured as a marker of leukocyte activation. Intracellular production of H_2_O_2 _in monocytes during ischaemia-reperfusion injury reached statistical significance (p = 0.014), although similar trends were observed with neutrophils these did not reach statistical significance. CRP was measured to assess the inflammatory response following mild episodes of ischaemia-reperfusion injury and resulted in a significant increase in the CRP concentration (p =< 0.001). There were also increased plasma concentrations of D-dimer and a trend towards elevated vWF levels, which were measured as markers of coagulation activation and endothelial damage respectively. Although significant changes in D-dimer concentrations were observed during ischaemia-reperfusion injury (p = 0.007), measurement of the vWF did not reach statistical significance.

**Conclusion:**

Tourniquet induced forearm ischaemia-reperfusion injury results in increased adhesiveness, trapping and activation of leukocytes. We report that, even following a mild ischaemic insult, this leukocyte response is immediately followed by evidence of increased inflammatory response, coagulation activity and endothelial damage. These results may have important implications and this pilot study may lead to a series of trials that shed light on the mechanisms of ischaemia-reperfusion injury, including potential points of therapeutic intervention for pathophysiological conditions.

## Background

The vascular endothelium is a major structural and functional component of all tissues. Endothelial cells are localised between the intravascular and extravascular spaces, and these cells play an important role in regulating vascular homeostasis. The endothelium regulates blood coagulation, blood flow, and various inflammatory processes such as controlling leukocyte migration, adhesion and activation [1].

Phagocytic leukocytes are components of the non-specific immune system. They are capable of phagocytosis and destroy damaged tissue cells and invading pathogens such as bacteria. Monocytes and neutrophils are examples of phagocytic leukocytes, with neutrophils being considered as the 'chief' phagocytic leukocyte. Both monocytes and neutrophils have been implicated to play a key role in the development of ischaemia-reperfusion injury, where they are intrinsically involved in leukocyte-endothelial cell interactions [2].

Ischaemia-reperfusion injury occurs in diseases such as ischemic heart disease, and during surgical procedures, which involve the application of a tourniquet, such as knee arthroplasty and total knee replacement [3-7]. During ischaemia-reperfusion injury it can be appreciated that interactions between the phagocytic leukocyte and endothelium involve the expression of various adhesion molecules. Specific adhesion molecules important in mediating adhesive interactions include CD62L (L-selectin) and CD11b (Mac-1) on monocytes and neutrophils. These bind to their corresponding counter-receptors to facilitate leukocyte-endothelial cell interactions [8-10].

Ischaemia-reperfusion injury causes activation of monocytes and neutrophils and adhesion of these cells to the endothelium (trapping) [11,12]. This results in the production and release of reactive oxygen intermediates (ROIs), such as hydrogen peroxide, by activated leukocytes. These cause endothelial dysfunction which itself accelerates the atherosclerotic process which might lead to its final complication resulting in myocardial infarction, stroke and peripheral vascular disease [13-20].

A previous study has recently demonstrated the role of leukocytes in damage to the vascular endothelium during ischaemia-reperfusion injury. This investigation verified leukocyte involvement during ischaemia-reperfusion injury, and provided evidence of increased endothelial cell damage, following relatively short periods of reperfusion [4]. The present report consists of a human study that employed an adapted model of ischaemia-reperfusion injury. It aimed to assess the role of leukocytes during this process, as well as to investigate the inflammatory and coagulation response. This determined whether sustained endothelial damage is incurred, following mild ischaemia, together with reperfusion measured at various time intervals.

## Methods

### Subject volunteers

Ethical approval for this pilot analysis study was received from the local research ethics committee (North Wales Central Research Ethics Committee, Reference Number – 05/WNo02/26). Ten healthy human volunteers were selected upon completing a health questionnaire to exclude individuals with cardiovascular disease such atherosclerosis and inflammatory disorders such as arthritis, and were recruited after informed consent (5 males and 5 females, mean age = 43).

### Blood collection and cell counting

Venous blood samples were collected into vacutainers containing di-potassium ethylene diamine tetra-acetic acid (EDTA) (1.5 mg/ml), tri-sodium citrate (0.11 mol/l) and serum clot activator (Greiner Bio-one, UK). Full blood counts were performed using a Coulter^® ^MicoDiff^18 ^automated cell counter (Beckman Coulter, UK).

### Model of tourniquet-induced forearm ischaemia-reperfusion injury

This model provided an adapted method of tourniquet-induced forearm ischaemia-reperfusion injury. During this study all subject volunteers were extubated prior to commencing the investigations with an 18GA cannula (BD Venflon™, Sweden), which was inserted into the ante-cubital fossa of the experimental arm. This was as a control measurement for that particular individual.

A sphygmomanometer was then placed around the upper experimental arm and inflated to approximately 20–40 mmHg for ten minutes as described by others [4,21-23]. This procedure reduced blood flow to the arm (ischaemia), following which time a blood sample was taken.

The cuff was then removed to allow full blood flow to the arm (reperfusion). Further blood samples were then collected, by means of the cannula at various time intervals (5, 15, 30 and 60 minutes). After collection of the 60 minute blood sample, the cannula was removed. Twenty four hours later a final blood sample was collected from the experimental arm by venepuncture.

### Preparation of cell suspensions

Purified neutrophils and mononuclear cell suspensions were prepared by density gradient sedimentation on ficoll hypaque solutions as described by Lennie *et al*, (1987) [24]. Following isolation, cells were re-suspended in phosphate buffered saline (PBS) supplemented with di-potassium EDTA (1.5 mg/ml) to yield a final cell count of 2 × 10^6 ^cells/ml. All chemicals were supplied by Sigma-Aldrich, UK.

### Measurement of cell surface expression of CD62L

**Figure 1:**
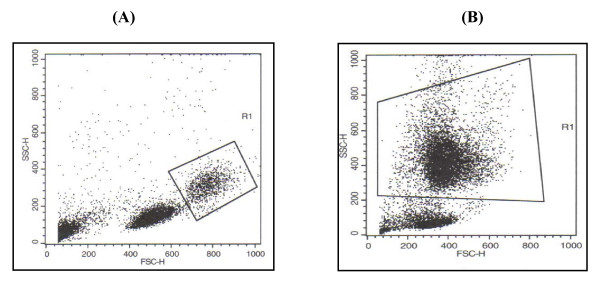
**Gating of phagocytic leukocyte sub-populations during flow cytometric analyses**. Gates were adjusted so that the percentage of cells analysed were identical to those identified using a Coulter^® ^MicroDiff^18 ^apparatus. Lymphocytes, red blood cells and debris were excluded from defined gates [4]. Leukocyte subpopulations were selected by assignment of gates normally associated with (A) monocytes and (B) neutrophils [36]. All flow cytometric analyses were performed using a Becton and Dickenson FACSCalibur flow cytometer.

The monoclonal antibodies used were mouse anti-human CD62L (MCA1076F) and isotype-matched control IgG2b (MCA691F) and were purified immunoglobulin/fluororescein isothiocyanate (Ig/FITC) conjugates (AbD Serotec Ltd., U.K.). Following isolation of leukocyte subpopulations and adjustment of concentration (2 × 10^6 ^cells/ml), 10 μl of the monoclonal antibody (0.1 mg/ml) was added to 100 μl of the appropriate cell suspension. These were incubated at room temperature for 30 minutes, prior to assay analysis using flow cytometry of gated monocytes and neutrophils (Figure 1).

### Measurement of cell surface expression of CD11b

The monoclonal antibodies used were mouse anti-human CD11b (MCA551F) and isotype-matched control IgG1 (MCA928F) and were purified immunoglobulin/fluororescein isothiocyanate (Ig/FITC) conjugates (AbD Serotec Ltd., U.K.). Following isolation of leukocyte subpopulations and adjustment of concentration (2 × 10^6 ^cells/ml), 10 μl of the monoclonal antibody (0.1 mg/ml) was added to 100 μl of the appropriate cell suspension. These were incubated at room temperature for 30 minutes, prior to assay analysis using flow cytometry of gated monocytes and neutrophils (Figure 1).

### Measurement of intracellular hydrogen peroxide production

Cells were isolated and intracellular H_2_O_2 _production was assessed by adaptation of a technique previously described by Bass *et al *(1983) [25]. The assay was based on the oxidation by H_2_O_2 _of non-fluorescent 2', 7'-dichlorofluoroscin diacetate (DCFH-DA) to stable and fluorescent dichlorofluoroescein. H_2_O_2 _production was assessed in cells using a fixed volume of 0.5 ml cell suspension (2 × 10^6 ^cells/ml) mixed with 0.5 ml DCFH-DA (20 μM) in PBS. Cells were incubated in the dark, at 37°C for 30 minutes before immediate measurement using flow cytometry of gated monocytes and neutrophils (Figure 1).

### Measurement of C-reactive protein (CRP)

Measurement of C-reactive protein was performed using an ILAB 600 clinical chemistry analyser (Instrumentation Laboratory, UK). Highly sensitive CRP was measured using the Quantex CRP plus kits which were supplied by Bio-kit (Spain) and involved using a turbidimetric assay as previously described by Price *et al *(1987) [26].

### Measurement of plasma concentration of D-dimer and von Willebrand Factor (vWF)

Blood samples were collected into tri-sodium citrate tubes and were centrifuged at 1500 *g *for 10 minutes within 4 hours of blood collection. Plasma was removed and stored at -30°C. Plasma concentrations of D-dimer and vWF were measured by a two step enzyme immunoassay sandwich method, with a final fluorescent detection as described by others [27,28]. Measurement of these parameters was performed using a Mini-Vidas automated immunoassay system that uses ELFA (Enzyme-Linked Fluorescent Assay) technology. The Mini-Vidas system and immunoassay kits were supplied from Biomerieux, UK.

### Statistical analysis

During this convenience pilot analysis study results are presented as mean ± SEM; n indicates the number of participants in the study. Changes in the measured parameters during ischaemia and reperfusion were determined by repeated measures analysis of variance (ANOVA – using the sweeping by treatment method) and the paired *t *test. Statistical significance was accepted when p ≤ 0.05. All statistical analysis was performed using Minitab Release 13 software package (Minitab Ltd, UK).

## Results

### Effect of tourniquet induced forearm ischaemia-reperfusion injury on CD62L (L-selectin) and CD11b cell surface expression of monocytes and neutrophils (n = 10)

**Figure 2:**
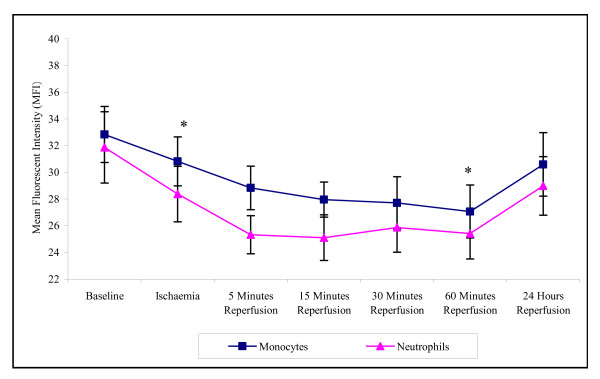
**Effect of tourniquet induced forearm ischaemia-reperfusion injury on CD62L cell surface expression of monocytes and neutrophils**. The results are expressed as mean fluorescent intensity (MFI) and represent the changes in the CD62L (L-selectin) cell surface expression of monocytes and neutrophils during the experimental stages of ischaemia-reperfusion injury. The points represent mean ± SEM, p = 0.006 (monocytes) and p =< 0.001 (neutrophils), as determined by ANOVA. *p =< 0.05 Baseline vs ischaemia and 60 minutes reperfusion for both monocytes and neutrophils, n = 10.

During the experimental stages of ischaemia and reperfusion there was a significant effect on CD62L expression (p = 0.006 for monocytes and p =< 0.001 for neutrophils). This expression on both monocytes and neutrophils decreased from baseline to 60 minutes reperfusion (32.84 ± 2.84 – 27.07 ± 1.98 monocytes; 31.88 ± 2.67 to 25.42 ± 1.90 neutrophils) (Figure 2). The CD62L cell surface expression for both cell types returned towards basal levels following 24 hours reperfusion (30.06 ± 2.38 monocytes; 28.99 ± 2.19 neutrophils), although this was expressed at a lower level to that of baseline.

**Figure 3:**
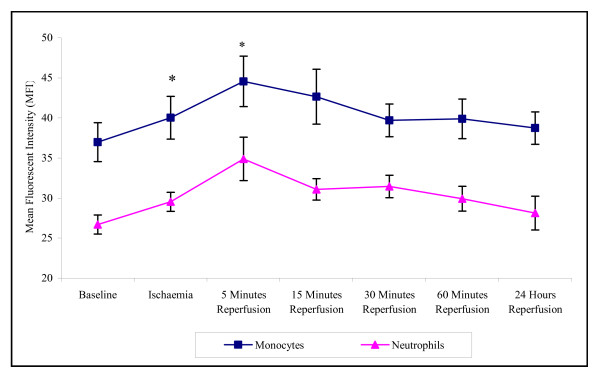
**Effect of tourniquet induced forearm ischaemia-reperfusion injury on CD11b cell surface expression of monocytes and neutrophils**. The results are expressed as mean fluorescent intensity (MFI) and represent the changes in the CD11b cell surface expression of monocytes and neutrophils during the experimental stages of ischaemia-reperfusion injury. The points represent mean ± SEM, p = 0.005 (monocytes) and p = 0.009 (neutrophils), as determined by ANOVA. *p =< 0.05 Baseline vs ischaemia and 5 minutes reperfusion for both monocytes and neutrophils, n = 10.

There was a significant effect of ischaemia and reperfusion on CD11b cell surface expression of monocytes (p = 0.005) and neutrophils (p = 0.009) (Figure 3). Results show that during 10 minutes ischaemia and 5 minutes reperfusion there is an increase in CD11b cell surface expression on monocytes and neutrophils. Following 24 hours reperfusion CD11b cell surface expression decreased and did not significantly differ from basal levels. The CD11b cell surface expression in monocytes was consistently higher than that seen in neutrophils.

### Effect of tourniquet induced forearm ischaemia-reperfusion injury on leukocyte trapping and activation (n = 10)

**Figure 4:**
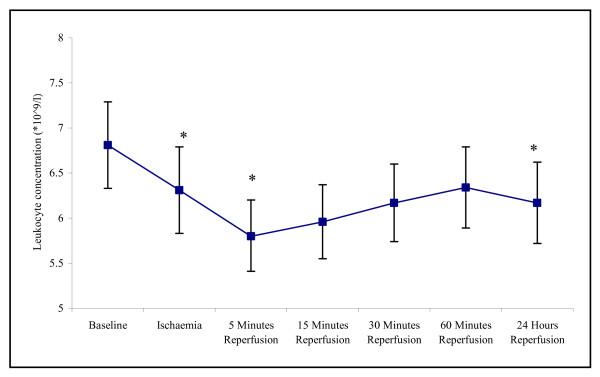
**Effect of tourniquet induced forearm ischaemia-reperfusion injury on total leukocyte concentration**. The results are expressed as total leukocyte concentration (10^9^/l). The points represent mean ± SEM, p =< 0.001, as determined by ANOVA. *p =< 0.05 Baseline vs ischaemia, 5 minutes and 24 hours reperfusion, n = 10.

There was evidence of increased leukocyte trapping during ischaemia and reperfusion, with the measured changes in the total leukocyte concentrations reaching statistical difference (p =< 0.001). The total leukocyte concentration decreased from 6.810 ± 0.48 at baseline to 5.82 ± 0.39 at 5 minutes reperfusion (p = 0.003) (Figure 4). Following 24 hours reperfusion the total leukocyte concentration increased back towards basal levels (6.17 ± 0.45) yet remained significantly lower (p = 0.027).

**Figure 5:**
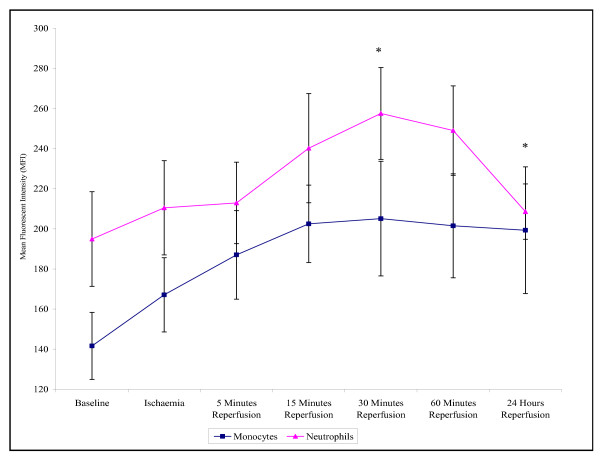
**Effect of tourniquet induced forearm ischaemia-reperfusion injury on intracellular H_2_O_2 _production of monocytes and neutrophils**. The results are expressed as mean fluorescent intensity (MFI) and represent the changes in the intracellular H_2_O_2 _production of monocytes and neutrophils during the experimental stages of ischaemia-reperfusion injury. The points represent mean ± SEM, p =< 0.001 for both monocytes and neutrophils, as determined by ANOVA. *p =< 0.05 Baseline vs 30 minutes (monocytes and neutrophils) and 24 hours reperfusion (monocytes), n = 10.

Both monocytes and neutrophils displayed an increase in the intracellular production of H_2_O_2 _from baseline up to 30 minutes reperfusion (141.6 ± 16.7 – 205.1 ± 28.6 monocytes (p =< 0.001); 194.9 ± 23.6 – 257.5 ± 22.9 neutrophils (p =< 0.001). Following 24 hours reperfusion the intracellular production of H_2_O_2 _by monocytes and neutrophils decreased back towards basal levels, although these remained at higher measurements to that of baseline (199.3 ± 31.3 monocytes (p = 0.014); 208.6 ± 13.8 neutrophils (p => 0.05)) (Figure 5). Neutrophils also displayed increased intracellular production of H_2_O_2 _compared to monocytes.

### Effect of tourniquet induced forearm ischaemia-reperfusion injury on the inflammatory response, coagulation activity and endothelial damage (n = 10)

**Figure 6:**
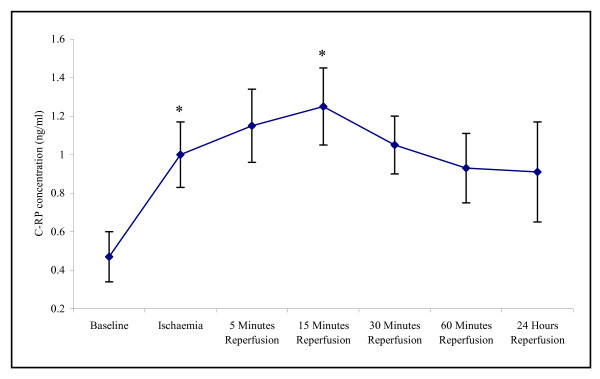
**Effect of tourniquet induced forearm ischaemia-reperfusion injury on C-reactive protein (CRP) concentration**. The results are expressed as CRP concentration (ng/ml). The points represent mean ± SEM, p =< 0.001, as determined by ANOVA. *p =< 0.001 Baseline vs ischaemia and 15 minutes reperfusion, n = 10.

Our study demonstrated that there was a significant effect of experimental ischaemia and reperfusion on the CRP (p =< 0.001) and D-dimer (p = 0.007). These parameters were measured as markers of the inflammatory response and coagulation activity respectively. The CRP concentration increased from baseline, during ischaemia, and peaking at 15 minutes reperfusion (0.47 ± 0.47 – 1.25 ± 0.20 p < 0.001). Following 24 hours reperfusion the CRP concentration decreased, although this was expressed at a higher concentration to that of basal levels (0.91 ± 0.26) (Figure 6).

**Figure 7:**
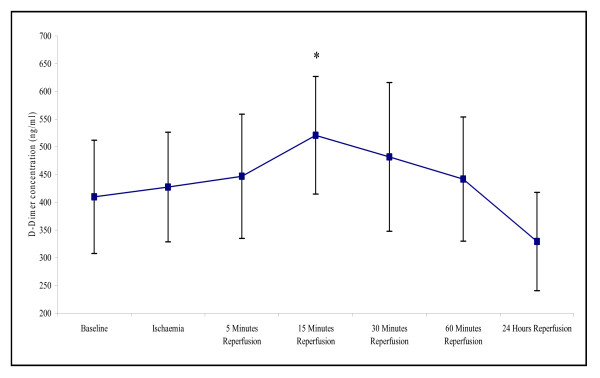
**Effect of tourniquet induced forearm ischaemia-reperfusion injury on D-dimer concentration**. The results are expressed as D-dimer concentration (ng/ml). The points represent mean ± SEM, p = 0.007, as determined by ANOVA. *p = 0.046 Baseline vs 15 minutes reperfusion, n = 10.

D-dimer concentration in the plasma was significantly affected by ischaemia and reperfusion (p = 0.007). Levels increased from baseline during ischaemia, and peaked at 15 minutes reperfusion (410 ± 102 – 520 ± 106; p = 0.046) (Figure 7). Following 24 hours reperfusion the D-dimer concentration decreased, although this was present at a slightly lower concentration (329.5 ± 88.6) to that of basal levels.

**Figure 8:**
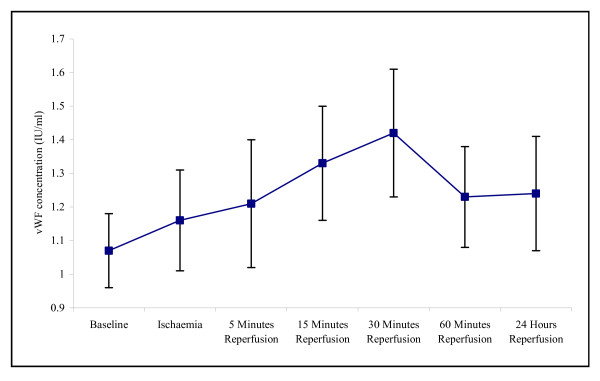
**Effect of tourniquet induced forearm ischaemia-reperfusion injury on vWF concentration**. The results are expressed as vWF concentration (ng/ml). The points represent mean ± SEM, p = 0.687, as determined by ANOVA, n = 10.

Host cell damage during an episode of ischaemia and reperfusion was assessed via measurement of vWF concentration, which is an established marker of endothelial damage [29]. During this study, changes in vWF concentration during ischaemia and reperfusion resulted in an increase in vWF concentration from baseline and peaking at 30 minutes reperfusion (1.07 ± 0.11 – 1.42 ± 0.19) (Figure 8). Following 24 hours reperfusion the vWF concentration decreased, although this was expressed at a higher concentration to that of basal levels (1.24 ± 0.17). However, none of these changes reached statistical difference.

## Discussion

An important aspect of this study was to provide a better understanding of the mechanisms by which phagocytic leukocytes are involved in ischaemia-reperfusion injury. Using a model of mild ischaemia-reperfusion injury in normal humans, we established that monocytes and neutrophils analysed *ex vivo *showed evidence of increased adhesion, trapping and activation. This was associated with in increased inflammatory response, coagulation activity and a trend towards sustained endothelial damage.

It can be appreciated that the adhesion and transendothelial migration of leukocytes into the surrounding tissues are crucial steps in inflammation, immunity, atherogenesis, and during ischaemia-reperfusion injury [4,30-32]. The present study was designed to ascertain whether ischaemia-reperfusion injury in normal individuals results in changes in the cell surface expression of the CD62L and CD11b adhesion molecules, and to specifically assess the leukocyte adhesion cascade in response to episodes of mild ischaemia and subsequent reperfusion. During this investigation there was evidence of increased shedding of CD62L from the cell surface of both monocytes and neutrophils, and our results support the evidence that CD62L plays a key role during the early stages of the leukocyte adhesion cascade, which facilitates leukocyte adhesion to the endothelium.

This was supported by the up-regulation of the CD11b cell surface adhesion molecules by both monocytes and neutrophils. This suggests that during ischaemia-reperfusion injury CD11b expressed on the cell surface of phagocytic leukocytes binds to counter-receptors, such as intercellular adhesion molecule-1 (ICAM-1), expressed on the surface of vascular endothelium, which facilitate leukocyte-endothelial interactions. In agreement with others, this increased expression of CD11b on leukocytes may therefore play a central role as the mechanism by which leukocyte adhesion, and consequently trapping in the microcirculation occurs during ischaemia-reperfusion injury [4].

To assess the degree of leukocyte trapping, as a result of adhesion of leukocytes to the endothelium, changes in the leukocyte concentration were measured in venous blood taken from the forearm. This was assessed by measurement of a full blood count using an automated cell counter as described previously [4]. Trapping of leukocytes to the endothelium would result in a decrease in the number of leukocytes leaving the arm [4]. Evidence of increased leukocyte trapping in blood vessels was supported by the reduction in monocyte and granulocyte cell numbers, and the increased shedding of CD62L and up-regulation in CD11b cell surface expression of monocytes and neutrophils. Collectively, it can be appreciated that in the present model these intrinsic events may play a key role during leukocyte adhesion to the endothelium of the microcirculation.

Increased leukocyte adhesion to the vascular endothelium during ischaemia-reperfusion leads to cell activation. Using tourniquet induced forearm ischaemia-reperfusion, we then investigated leukocyte activation by measuring the intracellular production of H_2_O_2 _by monocytes and neutrophils. Evidence of leukocyte activation during the experimental stages of tourniquet induced forearm ischaemia and reperfusion was supported by the increase in intracellular H_2_O_2 _production of monocytes and neutrophils. In addition, during leukocyte activation it can be appreciated that further bioactive material, such as other ROIs and proteolytic enzymes are released extracellularly [33,34]. Collectively, the actions of these degradative substances may potentially cause damage to host tissue in the current model. Measurement of the intracellular production of H_2_O_2_by phagocytic leukocytes may therefore provide a useful marker that could be applied to monitoring various diseases such as atherosclerosis and ischaemic heart disease, conditions where ischaemia-reperfusion injury can be appreciated to be an underlying process [4].

Another aspect of this study was to assess the inflammatory response, coagulation activity, and any endothelial damage that may be incurred, during tourniquet induced forearm ischaemia and various reperfusion time periods. Increased CRP concentration during these studies demonstrated that there was a mild inflammatory response during ischaemia and reperfusion, although clinically these may not be of significant concerns as all results are within the normal clinical reference range. It is proposed that the inflammatory changes observed during this study may be due to the release of cytokines such as interleukin-6 (IL-6) from the activated cells during mild episodes of ischaemia-reperfusion injury [35].

Changes in the D-dimer concentrations during this study demonstrate an increase in coagulation activity during the experimental stages of ischaemia and reperfusion injury. It has also been suggested that increased D-dimer concentrations may up-regulate IL-6 which in-turn may increase CRP levels [35]. Results from this particular study showed evidence of increased coagulation/fibrinolitic response and provides possible direction for further studies, which may involve the investigation of more parameters such as fibrinogen in response to episodes of ischaemia-reperfusion injury. Evidence of endothelial cell damage during ischaemia and reperfusion has previously been documented [3,4]. However, the results from our study compliment these earlier studies and provide further evidence of a trend towards increased and sustained host cell damage as shown by vWF levels during ischaemia-reperfusion injury. A possible explanation for this phenomenon is that there is an increased liberation of vWF from the storage organelles of the endothelium during ischaemia and reperfusion. Measurement of this parameter may therefore provide a useful clinical marker for monitoring various conditions, such as peripheral vascular disease and atherosclerosis, in which ischaemia-reperfusion injury can be appreciated to be an underlying process [3-7,35].

Ischaemia reperfusion injury occurs in diseases such as myocardial infarction, stroke and peripheral vascular disease and during surgical procedures such as aortic and orthopaedic surgery. The incidence of such diseases and surgery is high in the U.K. generally and even minor improvements in the treatment could have substantial cost benefits for the national health services and health benefits for the nation. If ischaemia-reperfusion injury does result in leukocyte activation and subsequent host tissue damage following episodes of ischaemia-reperfusion injury, this research may offer the potential of pharmacological intervention. For example, the production of recombinant soluble adhesion molecules or free radical oxygen scavengers may reduce leukocyte adhesion and activation respectively, which may lead to the prevention or early intervention of host tissue damage during diseases such as atherosclerosis and myocardial infarction, or surgery that involves the application of a tourniquet. This may ultimately improve patients' recovery under such circumstances.

## Conclusion

These studies reveal evidence that during even very brief periods of ischaemia and reperfusion in normal humans, monocytes and neutrophils are rapidly activated, accumulate within the vasculature and produce potent reactive oxygen intermediates. We report that even following a mild ischaemic insult, this leukocyte response is immediately followed by evidence of leukocyte activation and changes in inflammatory and coagulation markers. This study also provides an opportunity to investigate other markers of endothelial damage to support these findings. Further scientific research may also highlight potential points of therapeutic intervention for pathophysiological conditions. Indeed, the present investigation could provide a model in normal human subjects to the study the effects of therapeutic agents for diseases involving ischaemia-reperfusion injury.

## Competing interests

The author(s) declare that they have no competing interests.

## Authors' contributions

SFH carried out the isolation of leukocyte sub-populations, assessment of leukocyte adhesion, assessment of total leukocyte counts, assessment of leukocyte activation and the immunoassays. BDH performed assessment of the inflammatory response. GER participated in the design and collection of the blood sampling procedure. DRE and SSB provided general supervision and advised on the clinical implications. SFH, BDH and JFM supervised the study, participated in its design and coordination and drafted the manuscript. All authors read and approved the final manuscript.
